# Supplementary material for: Electrochemical Detection of H2O2 Using Bi2O3/Bi2O2Se Nanocomposites
Source: Nanomaterials (Basel). 2024 Oct 2;14(19):1592. doi: 10.3390/nano14191592 (PMC11478603; doi:10.3390/nano14191592)
Supplement: Supplementary file 1 [file nanomaterials-14-01592-s001.zip › nanomaterials-3185364-supplementary.pdf]

Supporting Information

Electrochemical Detection of H_2O_2 using Bi_2O_3/Bi_2O_2Se Nanocomposites

Pooja D. Walimbe, Rajeev Kumar *, Amit Kumar Shringi, Obed Keelson, Hazel Achieng Ouma and Fei Yan *

Department of Chemistry and Biochemistry, North Carolina Central University, Durham 27707, NC, USA; pwalimbe@eagles.nccu.edu (P.D.W.); ashringi@nccu.edu (A.K.S.); okeelson@eagles.nccu.edu (O.K.); houma@eagles.nccu.edu (H.A.O.)

* Correspondence: rkumar@nccu.edu (R.K.); fyan@nccu.edu (F.Y.)

1.1 Characterization of the materials

Powder X-ray diffraction (XRD) data were collected using a XRDynamic 500 diffractometer (Antor Paar). The instrument was equipped with a copper source, operated at a voltage of 45 keV and a power of 40 kW. UV–Visible-near-infrared spectra (UV-Vis-NIR) were collected on a Shimadzu UV–Visible-NIR spectrometer equipped with a Harrick Praying Mantis Diffuse Reflection Accessory. Spectra were referenced to a background of potassium bromide. The FEI XL30 SEM-FEG scanning electron microscope was used to examine the morphology and elemental mapping via energy-dispersive X-ray (EDX) analysis. XPS experiments were performed using a Physical Electronics VersaProbe III instrument equipped with a monochromatic Al k_α X-ray source ($h\nu$ = 1,486.6 eV) and a concentric hemispherical analyzer. Charge neutralization was performed using both low-energy electrons (<5 eV) and argon ions. The binding energy axis was calibrated using sputter-cleaned Cu (Cu $2p_{3/2}$ = 932.62 eV, Cu $3p_{3/2}$ = 75.1 eV) and Au foils (Au $4f_{7/2}$ = 83.96 eV). The Bi-compounds were charge referenced to Bi^{3+} at 159.0 eV in the Bi $4f_{7/2}$ spectra. Measurements were made at a takeoff angle of 45° with respect

to the sample surface plane. This resulted in a typical sampling depth of 3–6 nm (95% of the signal originated from this depth or shallower). Quantification was done using instrumental relative sensitivity factors (RSFs) that account for the X-ray cross-section and inelastic mean free path of the electrons. On homogeneous samples, major elements (>5 atom%) tend to have standard deviations of <3%, while minor elements can be significantly higher. The analysis size was ~200 μm in diameter. All electrochemical characterization were performed on Waverider 200 Bipotentiostat (Pine Research).

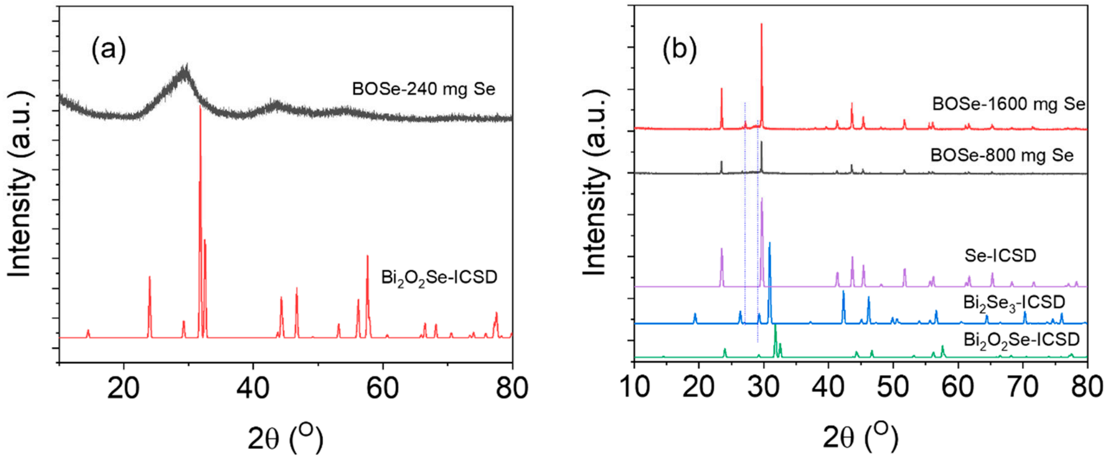

Figure S1. XRD of BOSe samples prepared using (**a**) 240 mg and (**b**) 800 and 1600 mg Se for the synthesis time of 18 h.

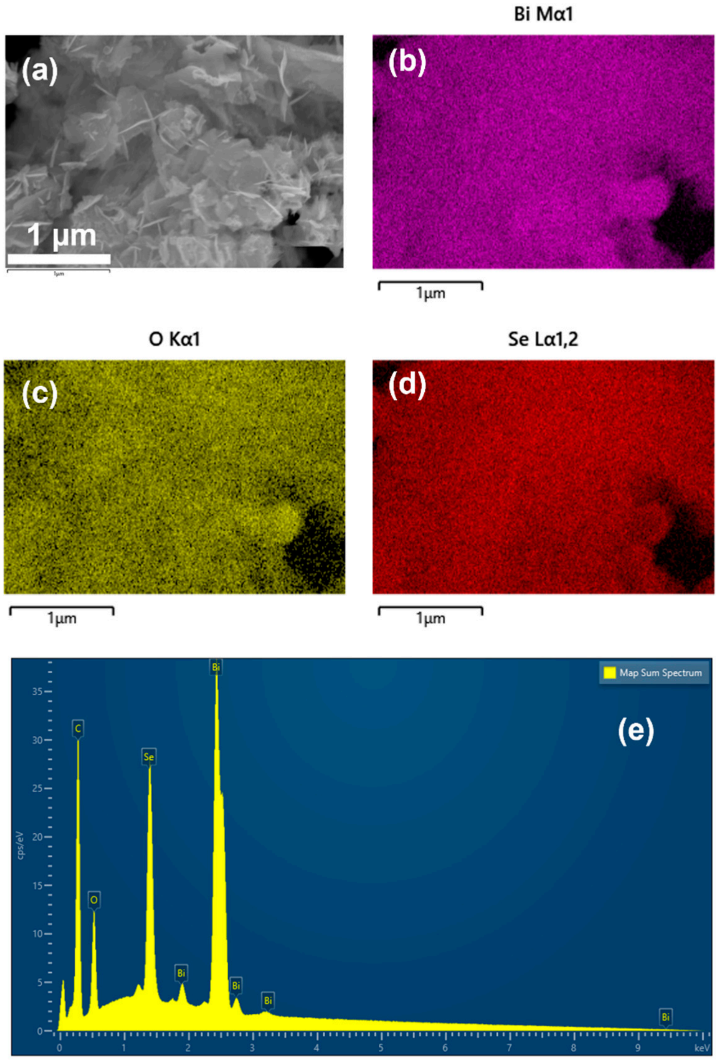

Figure S2. EDX analysis of the BOSe-6 h sample: **(a)** SEM image, elemental mapping for **(b)** Bi, **(c)** O, **(d)** Se, and **(e)** elemental scan.

Table S1. Elemental analyses of the BOSe-6 h sample from EDX.

**Map Sum Spectrum**							
Element	Signal Type	Line	Wt%	Wt% Sigma	Atomic %	Standard Name	Factory Standard
C	EDS	K series	23.28	0.08	70.21	C Vit	Yes
O	EDS	K series	6.22	0.04	14.08	SiO_2	Yes
Se	EDS	L series	12.20	0.05	5.60	Se	Yes

Bi	EDS	M series	58.30	0.10	10.11	Bi	Yes
Total			100.00		100.00		

Ratio of Bi: O: Se = 4:5:2

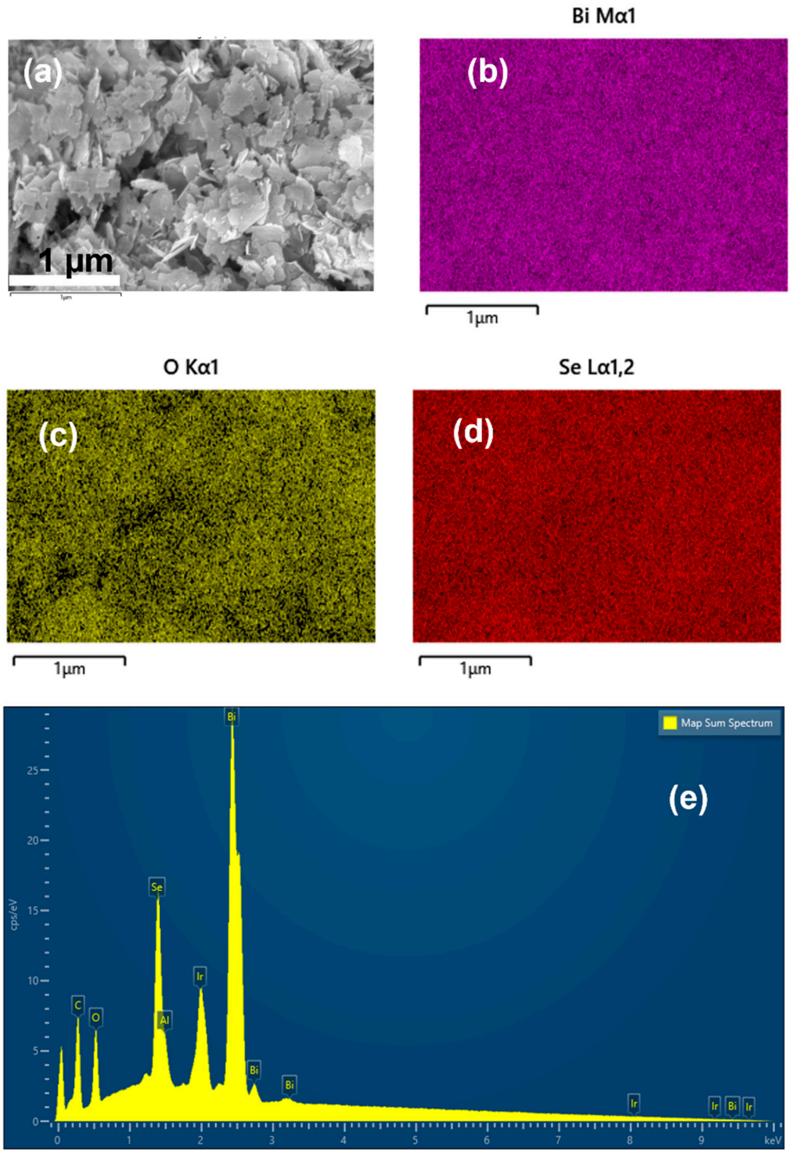

Figure S3. EDX analysis of the BOSe-7 days sample: **(a)** SEM image, elemental mapping for **(b)** Bi, **(c)** O, **(d)** Se, and **(e)** elemental scan.

Table S2. Elemental analyses of the BOSe-7 days sample from EDX.

**Map Sum Spectrum**							
Element	Signal Type	Line	Wt%	Wt% Sigma	Atomic %	Standard Name	Factory Standard
C	EDS	K series	10.01	0.09	49.04	C Vit	Yes
O	EDS	K series	5.27	0.05	19.39	SiO_2	Yes
Al	EDS	K series	1.24	0.04	2.71	Al_2O_3	Yes
Se	EDS	L series	11.53	0.08	8.60	Se	Yes
Bi	EDS	M series	71.94	0.12	20.26	Bi	Yes
Total			100.00		100.00		

**The presence of Al in the spectrum could be from the stub holder or from error estimation due to a peak overlap with Se.*

Ratio of Bi: O: Se = 2:2:1

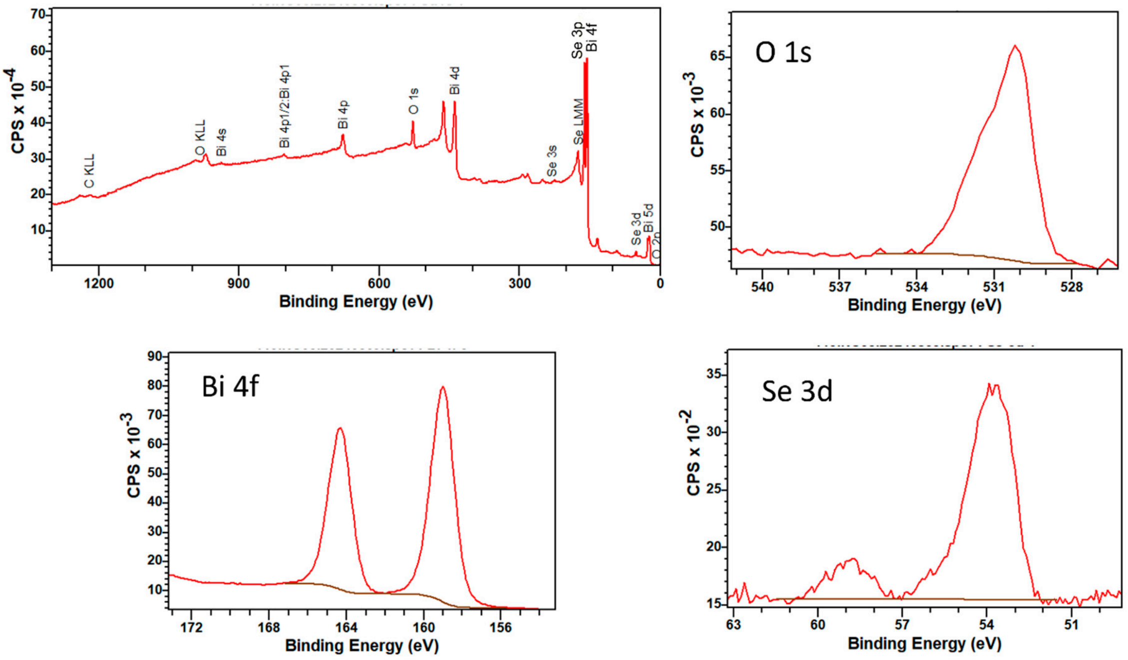

Figure S4. XPS analysis of the BOSe-6 h sample.

Table S3. Elemental analysis of the BOSe-6 h from XPS.

Element	Atom%
Bi	33.1
O	38.8
Se	7.7

Ratio of Bi: O: Se = 4.3: 5: 1

This suggests near a 1.075:1 ratio of Bi_2O_3:Bi_2O_2Se in this sample.

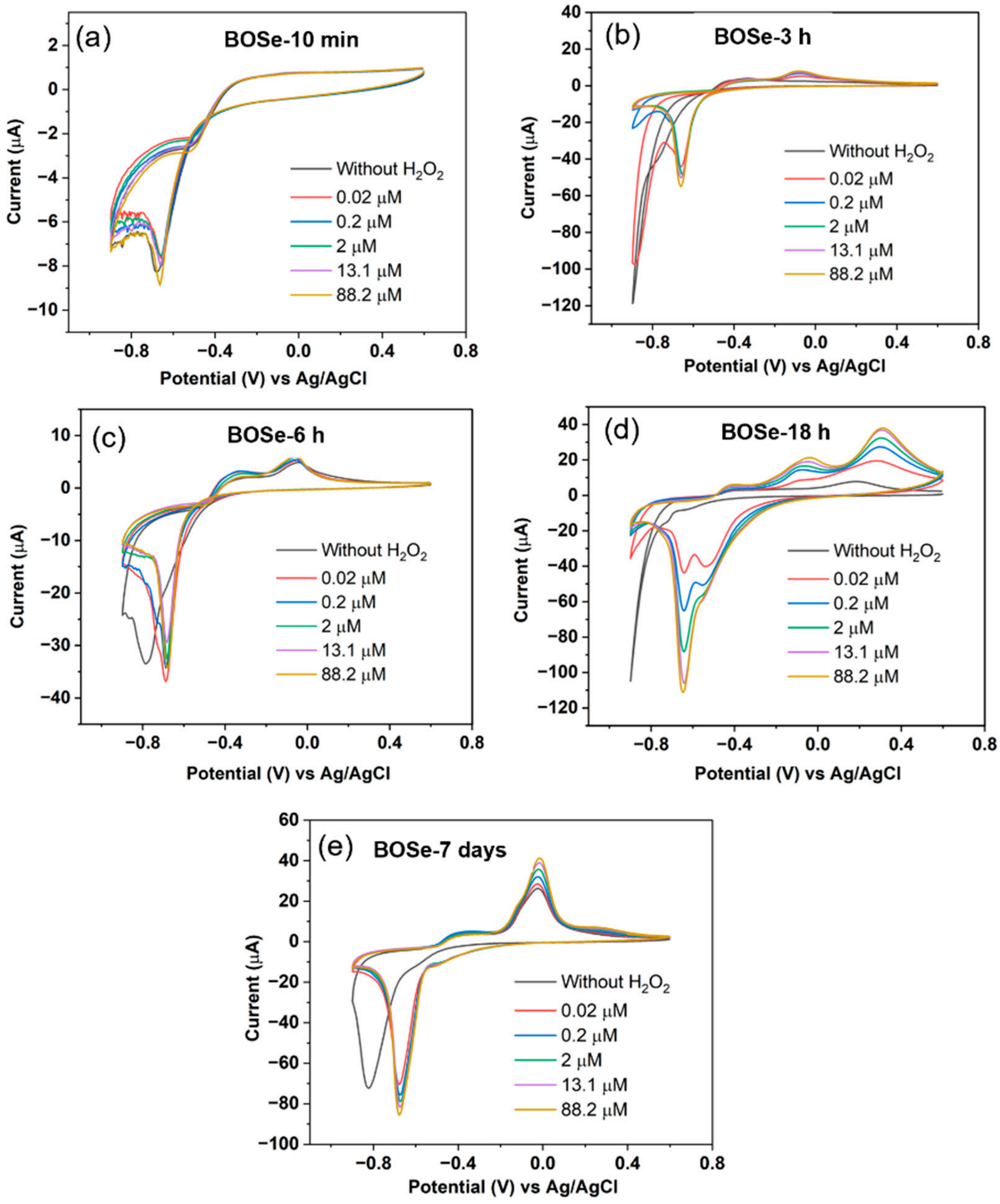

Figure S5. CV scans of Bi_2O_2Se with the synthesis time of (**a**) 10 min, (**b**) 3 hours, (**c**) 6 hours, (**d**) 18 hours, and (**e**) 7 days.

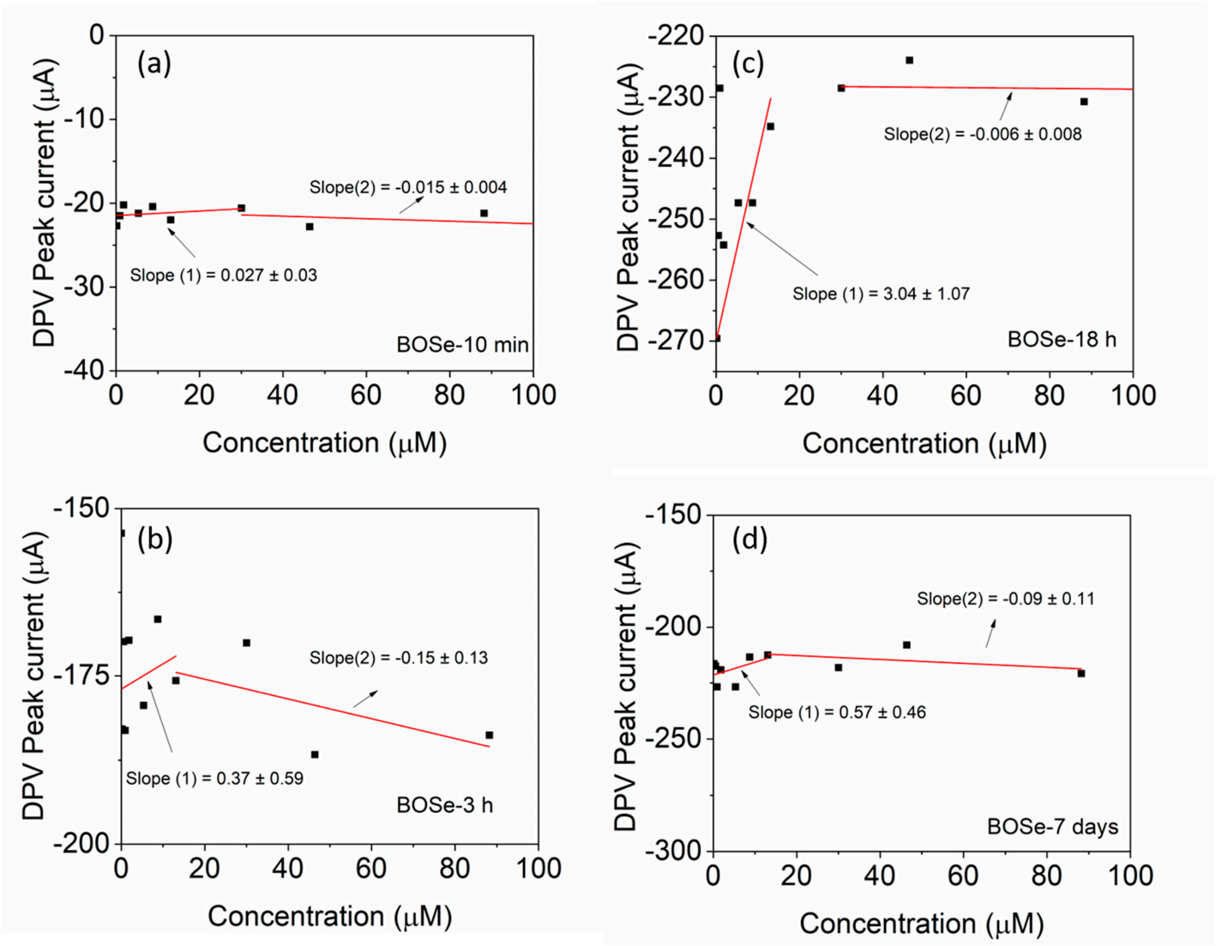

Figure S6. Calibration plots using DPV data for **(a)** BOSe-10 min, **(b)** BOSe-3 h, **(c)** BOSe-18 h, **(d)** BOSe-7 days. The black squares are the DPV peak data, while red lines are the linear fits.

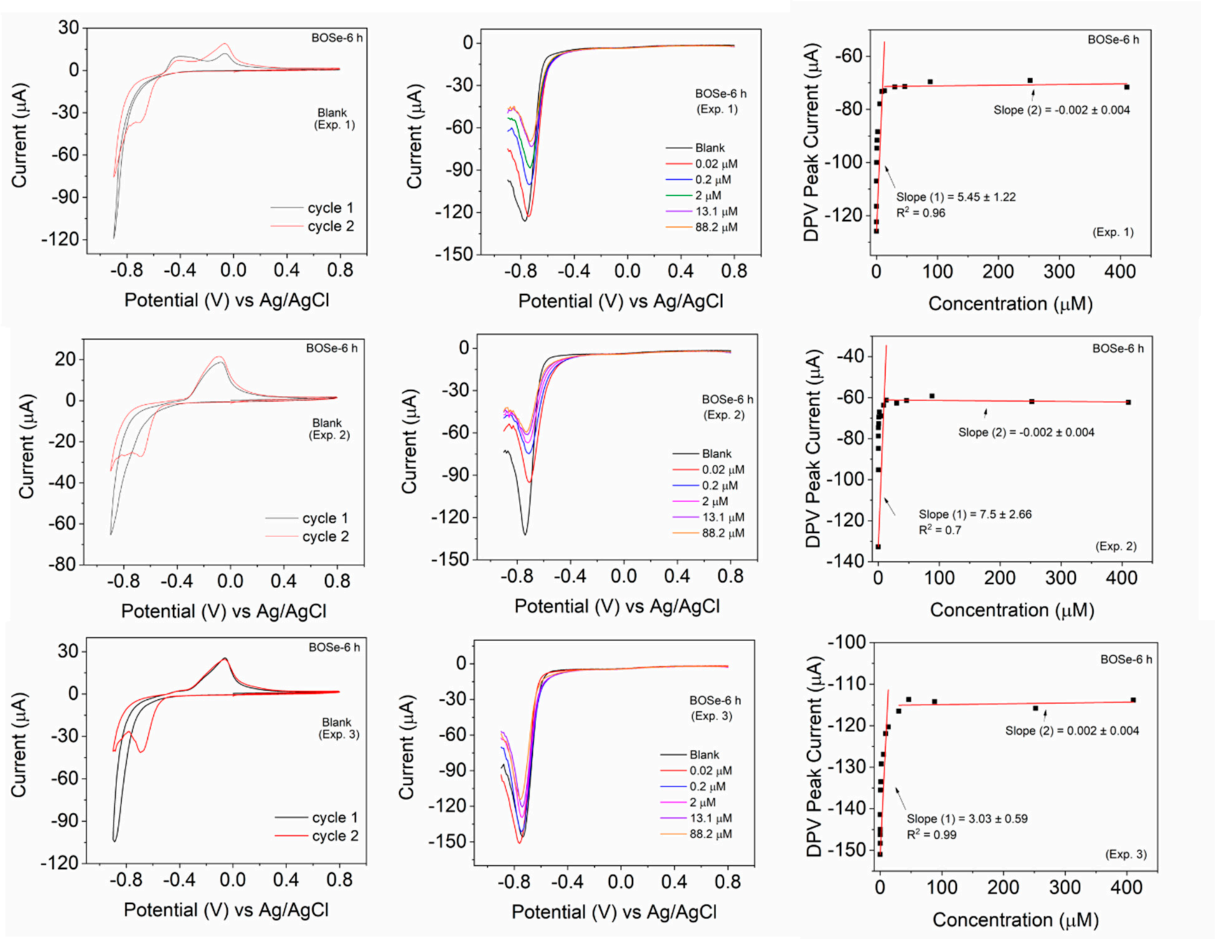

Figure S7. Reproducibility test using the BOSe-6 h sample, using a fresh electrode and electrolyte setup each time.

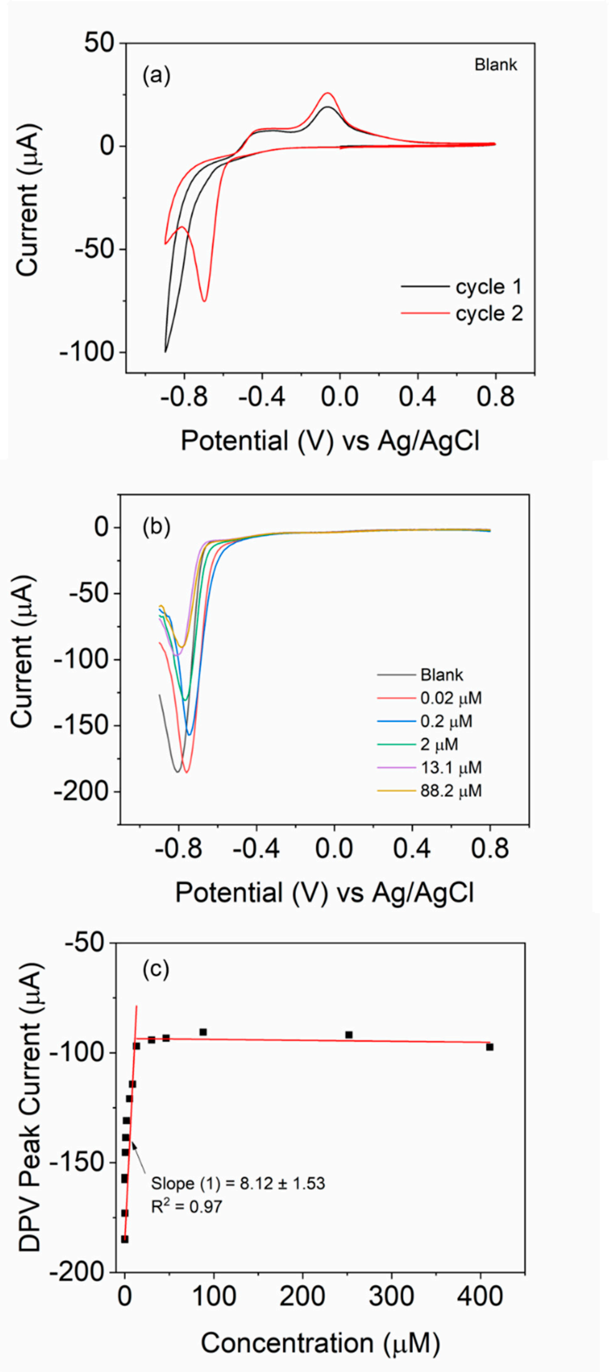

Figure S8. Electrochemical tests without Ar purging: (**a**) CV at 10 mv/s for the blank, (**b**) selective DPV scans with varying H_2O_2 amounts, and (**c**) calibration plot evaluated from DPV data.

Oxygen Reduction Reaction (ORR)

Rotating ring disk electrode (RRDE) studies were carried out in PBS solution saturated with oxygen. The rotation speeds for the electrode were varied from 400 to 2000 rpm. The H_2O_2 generated at the disk becomes oxidized at the ring, which is recorded as the ring current (I_R). The hydrogen peroxide selectivity (p) was calculated from the values of the disk current I_D, the ring current I_R, and the collection efficiency N using Equation (1):

$$p = \frac{2\frac{I_R}{N}}{I_D + \frac{I_R}{N}} \quad (1)$$

The current collection efficiency was found to be 0.222, which is evaluated from the RRDE studies carried out in nitrogen-saturated 1 mM hexa-amine-ruthenium (III) chloride dissolved in 0.1 M KCl. The hydrogen peroxide selectivity was then used to calculate the number of electrons transferred using Equation (2):

$$n = 4 - 2(p) \quad (2)$$

The BOSe-10 min sample showed the highest peroxide formation, followed by the BOSe-6 h sample.

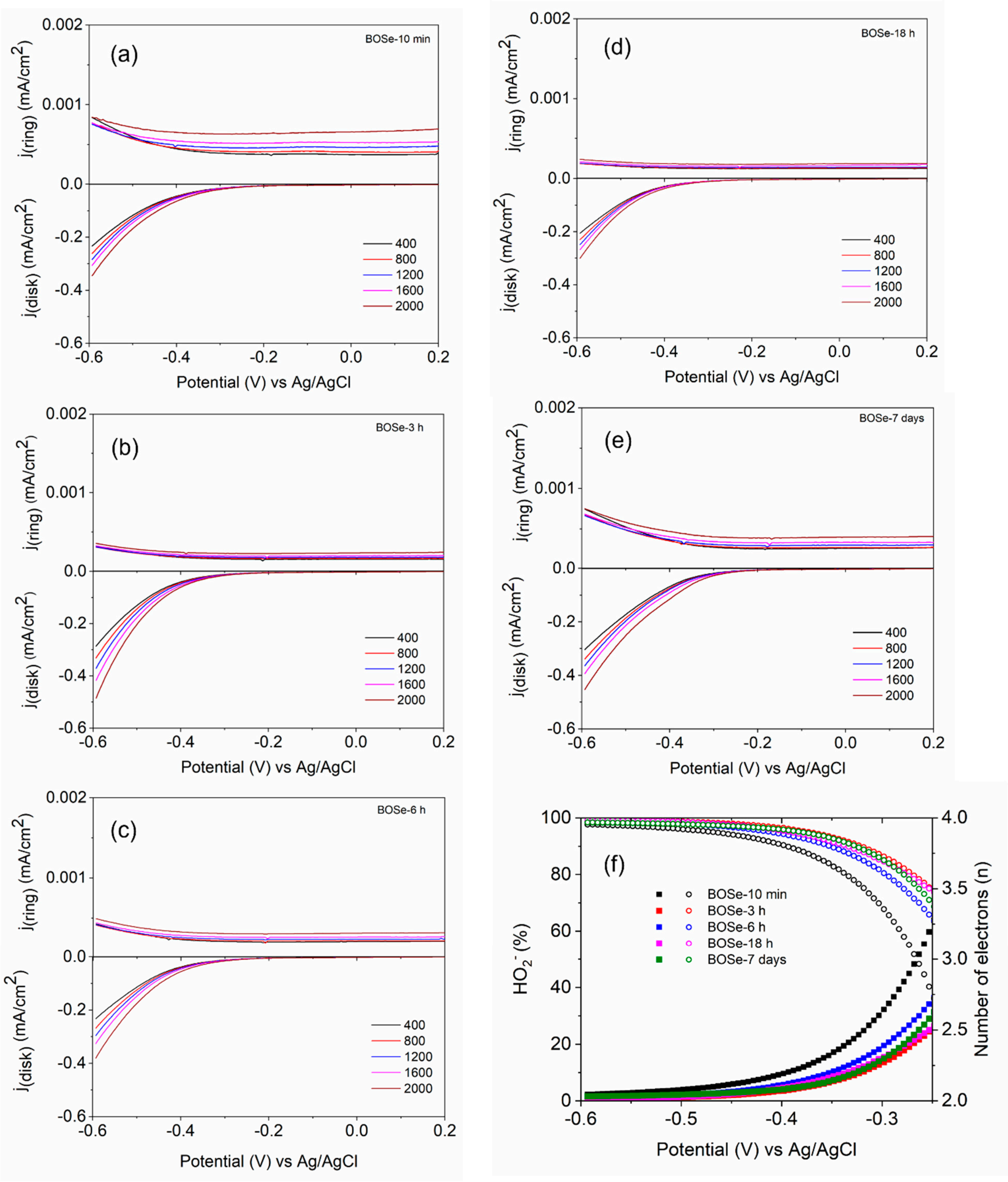

Figure S9. ORR using RRDE at different rotation speeds for (**a**) BOSe-10 min, (**b**) BOSe-3 h, (**c**) BOSe-6 h, (**d**) BOSe-18 h, and (**e**) BOSe-7 days. (**f**) Variation of HO_2^- % and transferred number of electrons as a function of potential.

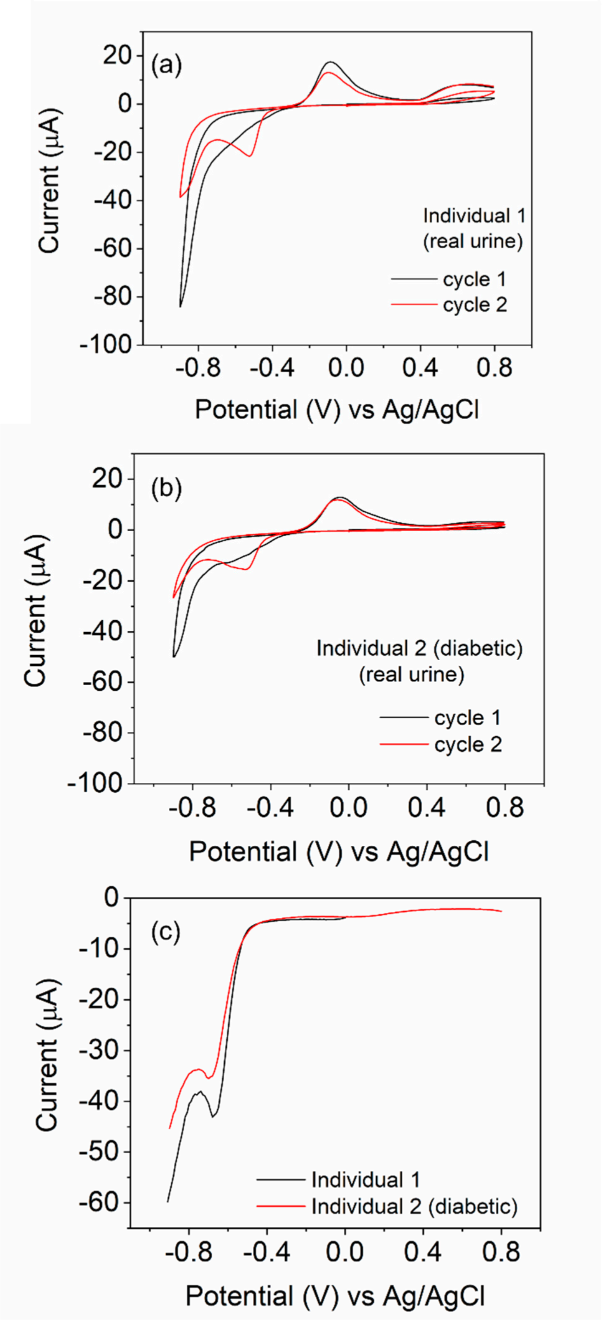

Figure S10. Electrochemical tests with 15 ml of real urine for two individuals using the BOSe-6 h sample. (**a**, **b**) CV and (**c**) DPV. The second individual is diabetic and shows a lesser current response, indicating more H_2O_2 in their urine. The direct quantification of the calibration in PBS could not be done, but this is more of a qualitative nature.
